# *Dnmt1* is required for early embryo development in the haplodiploid insect, *Bemisia tabaci* (Hemiptera: Aleyrodidae)

**DOI:** 10.1093/jisesa/ieaf077

**Published:** 2025-10-06

**Authors:** Emily A Shelby, Elizabeth C McKinney, Christopher B Cunningham, Alvin M Simmons, Allen J Moore, Patricia J Moore

**Affiliations:** Department of Entomology, University of Georgia, Athens, GA, USA; Department of Entomology, University of Georgia, Athens, GA, USA; Department of Entomology, University of Georgia, Athens, GA, USA; U.S. Department of Agriculture, Agricultural Research Service, U.S. Vegetable Laboratory, Charleston, SC, USA; Department of Entomology, University of Georgia, Athens, GA, USA; Department of Entomology, University of Georgia, Athens, GA, USA

**Keywords:** germ rudiment, reproduction, RNA interference, asexual, whitefly

## Abstract

The sweetpotato whitefly, *Bemisia tabaci* (Gennadius) (Hemiptera: Aleyrodidae), is a major economic pest that is difficult to manage with current strategies. New strategies will depend on better understanding the biology of whiteflies. For example, little is known about factors that affect primary sex ratio and embryological development in the haplodiploid system of *B. tabaci*, which may provide an entry point into new control strategies. In this study, we show that expression of DNA methyltransferase 1 (*Dnmt1*) is required for early embryogenesis in *B. tabaci*. First, we show that reduction of *Dnmt1* expression using RNA interference decreased the number of eggs laid and the viability of eggs but did not affect the adult sex ratio. We also identify key developmental stages during embryogenesis, which have been shown to be consistent in both sexes. Embryos produced from *dsDnmt1*-treated females failed to form a germ rudiment and had smaller sized nuclei, suggesting inhibition of the cell cycle early in development. Although the specific mechanism by which DNMT1 affects embryogenesis remains elusive, that is, whether the effect is methylation dependent or independent, our study provides insights into DNMT1’s function based on when and how DNMT1 is needed.

## Introduction

The sweetpotato whitefly, *Bemisia tabaci* (Gennadius) (Hemiptera: Aleyrodidae), is a globally important pest that causes billions of US dollars in damage to crops each year (Stansly and Naranjo 2010, Inoue-Nagata et al. 2016, Czosnek et al. 2017, Nwezeobi et al. 2020). *B. tabaci* is difficult to control as populations have developed resistance to most classes of insecticides (Horowitz et al. 2020). The economic importance of *B. tabaci* has led to an increase in the number of available technologies to make whitefly molecular studies more tractable such as sequenced genomes (Chen et al. 2016, Xie et al. 2017, Chen et al. 2019, Hussain et al. 2019), characterized methylomes (Catto et al. 2024, Cunningham et al. 2024), RNA sequencing datasets (Shen et al. 2023, Catto et al. 2024, Cunningham et al. 2024), transcriptomic and proteomic datasets (Yang et al. 2013), protocols for CRISPR (Heu et al. 2020) and RNA interference (RNAi; Dai et al. 2017, Gong et al. 2022, Jain et al. 2022, Shelby et al. 2023), life history summaries (Byrne and Bellows 1991, Aregbesola et al. 2020), and imaging methods (Luan et al. 2018, Bondy and Hunter 2019a, Shelby et al. 2023). Despite the availability of resources, aspects of whitefly biology, including those that might play a role in its success as a pest, continue to need further study, given its continuing pest status (Shelby et al. 2020).

One area that is surprisingly sparse in the whitefly literature is embryonic development, including determinants of primary sex ratio. Because there are no published studies on embryogenesis in *B. tabaci*, it is currently unknown to what extent the timing of embryonic development is conserved with other Hemiptera. In *B. tabaci*, which utilizes a haplodiploid sex determination system, virgin mothers only produce male offspring (haploid), whereas mated females produce both males (haploid) and females (diploid). While most *B. tabaci* population dynamic studies report on the factors that influence adult sex ratios (Pascual and Callejas 2004, Cui et al. 2008, Crowder et al. 2010, Tsueda and Tsuchida 2011, Wang et al. 2012, Lü et al. 2014, Parrella et al. 2014, Sun et al. 2014, Cass et al. 2016, Xiao et al. 2016), little is known about factors that influence primary sex ratios. Often studies that report “embryonic lethal” phenotypes following pesticide application do not include the developmental mechanisms that are being disrupted (Ishaaya et al. 1988, Ishaaya and Horowitz 1992), let alone if they are causing differential mortality of the sexes during embryonic development (Bondy and Hunter 2019b).

A fruitful avenue for understanding *B. tabaci* embryonic development is investigating the role of maintenance of DNA methyltransferase 1 (DNMT1; Shelby et al. 2023). Although the importance of DNA methylation in insects is unclear (Bewick et al. 2017, Duncan et al. 2022), DNMT1, the enzyme associated with maintaining DNA methylation patterns after DNA replication, is vital for proper gametogenesis and embryogenesis (Zwier et al. 2012, Kay et al. 2018, Schulz et al. 2018, Bewick et al. 2019, Amukamara et al. 2020, Ventós-Alfonso et al. 2020, Washington et al. 2021, Arsala et al. 2022, Cunningham et al. 2023, Ivasyk et al. 2023, Shelby et al. 2023, Bidari et al. 2024). In *B. tabaci*, knockdown of the messenger RNA for *Dnmt1* through RNAi leads to reduced levels of *Dnmt1* mRNA and the DNMT1 protein (Shelby et al. 2023). This results in the production of fewer eggs as well as fewer eggs successfully hatching (Shelby et al. 2023). Although there was evidence that these eggs were abnormal before oviposition (Shelby et al. 2023), it is unclear whether the eggs failed to develop due to abnormalities during oogenesis that prevented the initiation of embryogenesis or if embryonic development was initiated but were prohibited from successfully progressing. Also, because previous experiments used exclusively virgin females, and thus all embryos examined were male, it is unclear if loss of DNMT1 function results in differential mortality of the sexes during embryonic development.

The aim of our study was to determine if there was a role for DNMT1 in embryogenesis in *B. tabaci*, and if that role was sex-specific or associated with mode of reproduction. First, we performed maternal RNAi followed by mating experiments and measured fecundity, viability, and sex ratios to determine if DNMT1 influences egg viability differences in mated or unmated females, and the sex ratio of offspring from mated females. We then investigated the phenotypes associated with *Dnmt1* RNAi treatment on embryonic development, such as changes in nuclei morphology. As part of those experiments, we developed a timeline of key events in *B. tabaci* embryogenesis to identify if differences in sex affected timing of embryonic development as well as the effect of *Dnmt1* knockdown. We see no major differences in eggs produced from virgin or mated females and identify the specific developmental stage where *Dnmt1* appears to be required.

## Materials and Methods

### Insect Rearing

All individuals used in our experiments were obtained from laboratory-reared MEAM1 *B. tabaci* colonies. These colonies were started from wild-caught populations collected from cotton fields in Tift County, Georgia, USA, in 2018 (McKenzie et al. 2020). In the laboratory, we reared the colonies on collard plants (*Brassica oleracea* var. *viridis*). We kept both *B. tabaci* and collards in a Percival incubator (Model #I-36VLC8) at 27 °C with a 14:10 h light:dark photoperiod and constant temperature and photoperiod throughout experiments. We began with virgin females 3 to 5 d postadult eclosion (PAE) for all experiments. To ensure the correct age and mating status, we removed newly eclosed females from nymph colonies within 24 h of adult eclosion. For experiments that required mated females, we paired virgin females 3 to 5 d PAE with males 3 to 5 d PAE.

### Maternal RNA Interference

We prepared double-stranded interfering RNA and performed the knockdown protocol according to Shelby et al. (2023). Briefly, we made DNA templates of *Dnmt1* (our RNAi knockdown treatment) and *eGFP* (our RNAi control treatment) using PCR amplification with gene-specific primers and 500 ng/µl RNA (Table 1). We synthesized sense and antisense RNA in a single reaction using the Ambion MEGAscript RNAi kit (ThermoFischer Scientific, Waltham, Massachusetts, United States) following the manufacturer’s protocols. Following extraction and ethanol precipitation, we aliquoted the double-stranded RNA (dsRNA) and stored it at −80 °C until use.

**Table 1.** Primer sequences used for dsRNA synthesis

Gene	Sense primer	Antisense primer
*Dnmt1*	TCAATGATCATGATGAAAGGCCGCA	TGTCAGTGCTGACATTCCACACGGA
*eGFP*	CGAATTCACTAGTGATTTTACTTG	GCGGGAATTCGATTTGACC

We treated virgin females 3 to 5 d PAE with a dsRNA solution using an artificial feeding mechanism previously described in Shelby et al. (2023) for 24 h. The dsRNA feeding solution contained green food coloring (McCormick & Company, Baltimore, Maryland, United States). The food coloring allowed us to confirm if a female had fed on the solution. If the females fed on the solution, their abdomens would appear green when observed under a microscope. We only used females in these experiments which we confirmed had imbibed the dsRNA solution.

### Egg Collection, Development, and Adult Sex Ratios

Following feeding, we placed dsRNA-fed females on approximately 15-cm tall collard plants. As an RNAi control group, we placed untreated (not fed dsRNA) females 3 to 5 d PAE on 15-cm tall collard plants. Each plant harbored 50 to 100 females. For the experiments with mated females, we placed the females on 15-cm tall collard plants with an equal number of males. Whiteflies were sexed based on abdominal morphology.

We checked plants for eggs every 6 h for 5 d. If eggs were present, we removed adults from that plant and placed the adults on a new plant. We checked plants for eggs every 6 h. We recorded the number of eggs from mated untreated, *dsDnmt1*-treated, and *dseGFP-*treated females daily.

For fecundity assays, viability assays, and adult sex ratio assays, we allowed eggs to develop into adults on the collard plants. Similar to what is described in Shelby et al. (2023), we recorded the number of eggs deposited and the number of eggs that hatched. Data were collected from 30 clutches: 5 clutches from untreated mated females, 5 clutches from untreated virgin females, 5 clutches from *dseGFP*-treated mated females, 5 clutches from *dseGFP*-treated virgin females, 5 clutches from *dsDnmt1*-treated mated females, and 5 clutches from *dsDnmt1*-treated virgin females. We maintained individuals on the same plant from hatching until adult emergence. After adults emergence, we removed all adults from plants and recorded the numbers of males and females.

To establish key developmental timelines during embryogenesis, we sampled eggs from virgin and mated untreated females at approximately 6, 12, 18, 24, 48, 72, and 84 h postoviposition (PO). We defined the developmental stages based on descriptions of embryogenesis in *Oncopeltus fasciatus* (Dallas) (Panfilio et al. 2006, Chipman 2017) and *Acyrthosiphon pisum*. (Harris) (Miura et al. 2003). To identify the effects of *Dnmt1* knockdown on embryogenesis, we collected eggs from mated and virgin females that were either treated with *dsDnmt1* or *dseGFP*. For knockdown assays, we used eggs at approximately 6, 18, and 24 h PO as these were considered critical times for development. We removed collard leaves with whitefly eggs and either immediately removed the eggs from the leaves or kept the eggs on the leaves until they were the appropriate age. We wrapped the leaves in a damp paper towel and placed them in a sealed Petri dish in the incubator at 27 °C to prevent desiccation.

### Image Acquisition and Analysis

We removed eggs from collard leaves using a probe mounted with a minuten pin. After removing the eggs from the leaves, we dechorionated the eggs by soaking them in 5% sodium hypochlorite for 15 minutes. We processed eggs from the same plant and collection event together in a centrifuge tube cap. Following the removal of the chorion, we washed embryos in PBS and incubated in 32% paraformaldehyde overnight (approximately 16 h) at 2 °C. To visualize DNA, we used 1 µl of 0.5 µg/ml DAPI in PBS (ThermoFisher Scientific, Waltham, Massachusetts, United States). We mounted the stained embryos with Mowiol 4-88 mounting medium (Sigma-Aldrich, St Louis, Missouri, United States). We imaged the embryos with a Zeiss LSM 880 Confocal Microscope (Zeiss). Z-stack maximum projection images were taken. Only global image adjustment features (such as brightness and contrast) were used. All confocal images were falsely colored.

We successfully processed and analyzed images from a total of *N *= 149 embryos; this included *N *= 100 embryos from untreated mated females, *N *= 14 embryos from virgin *dseGFP*-fed females, *N *= 10 embryos from virgin *dsDnmt1*-fed females, *N *= 15 embryos from mated *dseGFP-*fed females, and *N *= 10 embryos from mated *dsDnmt1*-fed mated females. For nuclei measurements, we used the ImageJ measuring software (Version 1.54i by FIJI). Each value represents the average nuclei size for each embryo. For embryos with less than 30 nuclei, we measured all nuclei for the average. For embryos with more than 30 nuclei, we measured 30 randomly selected nuclei for the average. In embryos that began forming a germ rudiment, we only measured nuclei from embryo-forming tissues.

### Statistical Analyses

We conducted an ANOVA, followed by a Tukey HSD post hoc test to compare the effect of RNAi treatment on the number of eggs produced. Previously, we performed an ANOVA to test the effects of both RNAi treatment and mating status on the number of eggs produced. However, only RNAi treatment was significant, and there was no effect of mating status or an interaction between the 2 factors. Therefore, we found it appropriate to pool data points across mating status level (Little and Hills 1978, Quinn and Keough 2002). For transparency, the data for mating status level are represented using points with different shapes and colors.

We conducted a repeated *G* test (goodness-of-fit test) to compare the effect of treatment and mating status on egg viability. We also conducted a repeated *G* test to compare the effect of treatment on adult sex ratio. Repeated *G* tests are appropriate when both variables are categorical and when experiments are repeated many times, eg when measuring survival (yes/no) or sex ratio (male/female) in multiple families (repeated experiment) (Sokal and Rohlf 1995, McDonald 2014). The use of repeated *G* tests allows us to test for variation among experiments, and account for this variation in testing for an overall effect in a pooled analysis (Sokal and Rohlf 1995, McDonald 2014). The repeated *G* test provides information on appropriateness of pooling by testing for heterogeneity while protecting against inflated degrees of freedom (Sokal and Rohlf 1995, McDonald 2014). For both replicated *G* tests, we first performed a *G* test of independence to give a heterogeneity *G* values. We then pooled data for *G* test analysis of RNAi treatment effects. We performed a *G* test to determine whether the proportion of embryos at a given developmental stage was equal between virgin and mated females.

We used an ANOVA followed by a Tukey HSD post hoc test to compare the effect of RNAi treatment and mating status on nuclei area. For data analysis, we used Base R in RStudio (RStudio Team 2023). We used the ggplot2 and tidyverse packages for data visualization (Wickman 2016).

## Results

### *Dnmt1* Knockdown Reduces Fecundity and Viability in Virgin and Mated Females

**Fig. 1.**
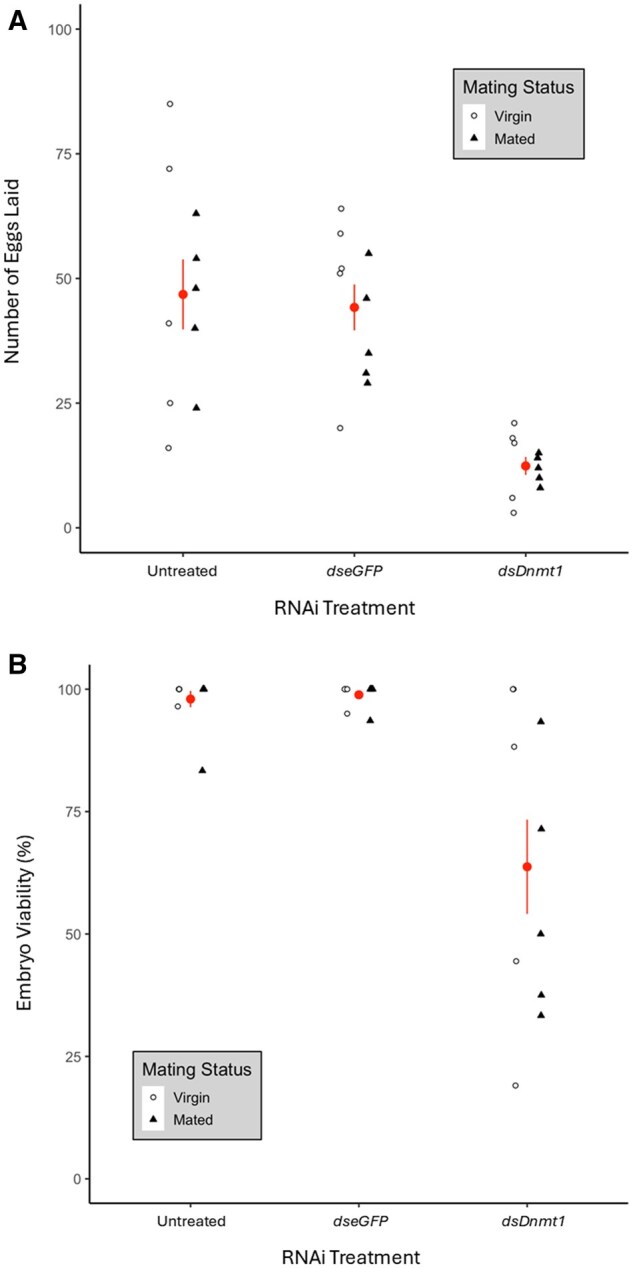
*Dnmt1* knockdown reduced fecundity and egg viability in virgin and mated *B. tabaci* females. A) Fecundity was reduced *dsDnmt1*-treated females. B) Eggs laid by *dsDnmt1*-treated females had reduced viability. The values are represented as mean ± SE (circle and bars, respectively) and as individual values (point). Mating status is indicated by point fill and shape. For each RNAi treatment, *N* = 10 cages with 100 females per cage.

RNAi treatment had a significant effect on the number of eggs laid (ANOVA; *F *= 14.92, df = 2,27, *P *< 0.001; Fig. 1A). There was no significant difference in the number of eggs laid between untreated and *dseGFP*-treated females (*P *= 0.93; Fig. 1A). Overall, *dsDnmt1*-treated females laid fewer eggs than untreated (Tukey HSD; *P *< 0.001) and *dseGFP*-treated (Tukey HSD; *P *< 0.001) females.

The *dsDnmt1* treatment significantly influenced egg viability compared to untreated and *dseGFP*-treated in both virgin (goodness-of-fit; *G *= 108.53, df = 2, *P *< 0.001) and mated (*G *= 72.74, df = 2, *P *< 0.001) females (Fig. 1B). Overall, egg viability was reduced by about a third in *dsDnmt*-treated females compared to untreated and *dseGFP*-treated females. In addition, control and *dsDnmt1* replicates displayed statistically significant heterogeneity (virgin untreated; *G *= 11.75, df = 4, *P *= 0.019; virgin *dsDnmt1*; *G *= 31.86, df = 4, *P *< 0.001; mated untreated; *G *= 13.69, df = 4, *P *= 0.008; mated *dsDnmt1*; *G *= 17.69, df = 4, *P *< 0.001). This heterogeneity was driven by 1 or 2 of the replicates in both the virgin and mated untreated females. In contrast, heterogeneity was not statistically significant in the *dseGFP*-treated females (virgin; *G *= 5.07, df = 4, *P *= 0.28; mated; *G *= 5.14, df = 4, *P *= 0.27). Moreover, the variation was far more extreme in the *dsDnmt1*-treated virgin and mated females compared to either untreated of *dseGFP* treatments (Fig. 1B), supporting a strong effect of the knockdown treatment.

### *Dnmt1* Knockdown Does Not Affect Adult Sex Ratio of Offspring

In contrast to egg viability, there was no statistically significant effect of RNAi treatment on sex ratio of offspring produced by mated females (*G *= 1.97, df = 2, *P *= 0.37; Table 2) and thus no evidence of differential effects of treatment on viability to adulthood. Again, there was significant heterogeneity among the untreated replicates (*G *= 23.53, df = 4, *P *< 0.001) and the *dseGFP* replicates (*G *= 30.45, df = 4, *P *< 0.001), but not among the *dsDnmt1* replicates (*G *= 3.66, df = 4, *P *= 0.45). This variation is likely to be random, given the differences were not unidirectional. In all the treatments, 100% of the offspring produced by virgin females were male as expected.

**Table 2.** Sex ratio of *Bemisia tabaci* adults in relation to the RNAi treatment group

RNAi treatment	Male	Female	Total
Untreated	122 (55%)	100 (45%)	222 (100%)
*dsDnmt1*	17 (50%)	17 (50%)	34 (100%)
*dseGFP*	76 (40%)	116 (60%)	192 (100%)
Total	215 (48%)	233 (52%)	448 (100%)

### Overview of Development in Embryos from Untreated Females

**Fig. 2.**
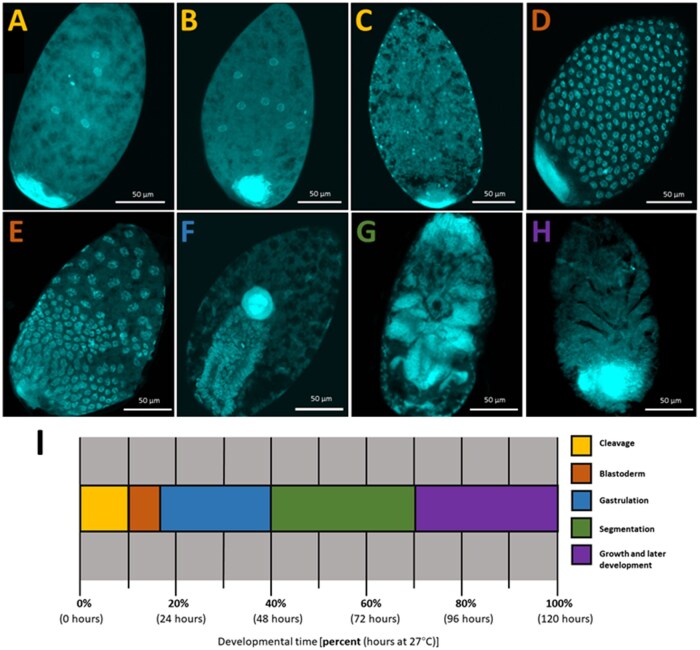
Illustration of key developmental stages during *B. tabaci* embryogenesis by DAPI nuclear staining: A to C) cleavage, D to E) blastoderm, F) gastrulation, G) segmentation, and H) growth and later development. I) A developmental timeline illustrating the onset and duration of stages examined in this study (color of letter denoting embryo image corresponds with the stage color on the timeline).

Like other hemipterans, the *B. tabaci* embryos developed as a syncytium; during the cleavage stage from 0 to 12 h PO, all nuclei are contained in a common cytoplasm (Fig. 2A to C and I). Syncytial cleavage began initially at the center of the egg around (Fig. 2A) and expanded outward toward the periphery (Fig. 2B). During this time, the nuclei divided synchronously, and the size of the nuclei varied as they rapidly progressed through the cell cycle (as seen in Fig. 2A to C). After reaching the periphery, the nuclei were regularly spaced and approximately the same size, suggesting that they were undifferentiated (Fig. 2D). During the blastoderm stage (12 to 18 h PO) (Fig. 2D and E, I), 2 cell populations began to segregate: blastoderm cells and extraembryonic cells (Fig. 2E). The blastoderm cells concentrated toward the posterior region near the bacteriocyte and began to form the germ rudiment (Fig. 2E). Gastrulation took place from 18 to 48 h PO (Fig. 2F and I). This stage began with the condensation of the germ rudiment and invagination toward the center (Fig. 2F). The bacteriocyte also moved toward the center (Fig. 2F). As a result of this movement, the embryo extended with the cephalic region at the posterior region of the egg. Segmentation occurred approximately 48 to 84 h PO (Fig. 2G and I). Cephalic and thoracic appendages were visible first (Fig. 2G), followed by abdominal segments which housed the bacteriocyte. For most of segmentation, the embryo was immersed in yolk. Subsequent stages of growth and development occurred until approximately 120 h PO when the embryo hatched as a nymph (Fig. 2H and I). These stages included elongation of appendages and the dorsal closure of the embryo (Fig. 2H). During this stage, visualization with fluorescent nuclear staining was disturbed, an expected artifact likely due to secretion of the cuticle. There was no difference in developmental timing based on mating status, as the proportion of embryos at a given developmental stage was equal between virgin and mated females (*G *= 0.44, df = 9, *P *= 1).

### The Effect of *Dnmt1* Knockdown on Early Embryogenesis

**Fig. 3.**
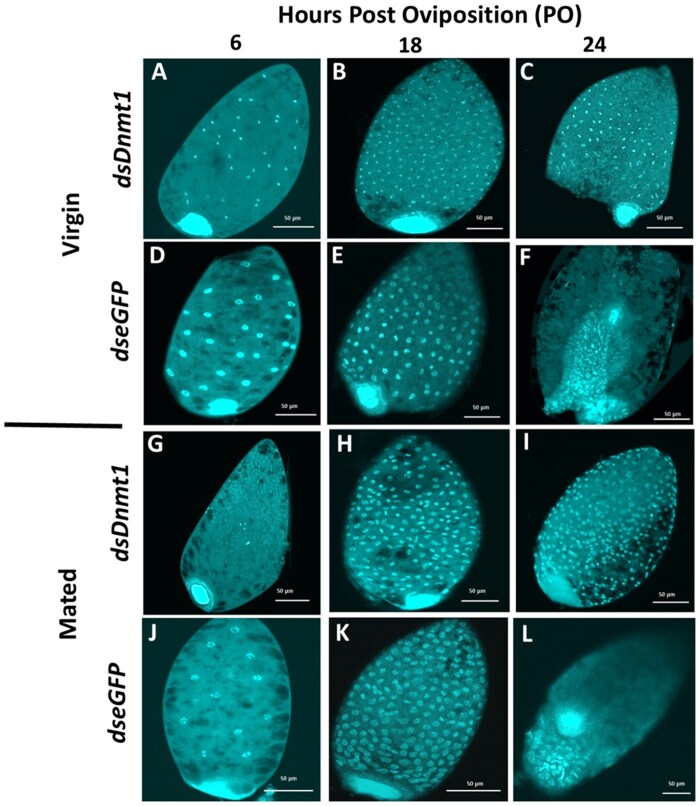
Knockdown of *Dnmt1* via maternal RNAi results in inability of *B. tabaci* to form a germ rudiment at 24 h postoviposition (PO). Images were taken at 6, 18, and 24 h PO of embryos from virgin A to F) and mated females G to L) and *dsDnmt1*-treated females A to C, G to I) and *dseGFP* treated D to F, J to L).

Maternal RNAi knockdown of *Dnmt1* produced lethal phenotypes in 70% of embryos. These embryos did not develop beyond the blastoderm stage (18 h PO) and failed to form a germ rudiment (Fig. 3). The remaining 30% of embryos did not have a knockdown phenotype, suggesting incomplete penetrance, possibly occurring due to individual differences in female feeding. At 24 h PO, embryos from virgin and mated *dsDnmt1*-treated females had similar knockdown phenotypes (Fig. 3C and I). In contrast, embryos from virgin and mated *dseGFP*-treated females proceeded to undergo gastrulation at 24 h PO (Fig. 3F and L). These results suggest that *Dnmt1* may be required for germ rudiment formation.

**Fig. 4.**
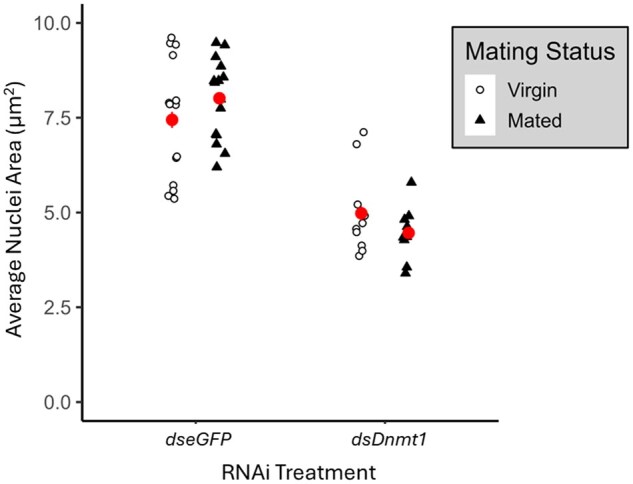
Loss of *Dnmt1* resulted in a smaller average nuclei area in embryos from virgin and mated *B. tabaci* females. The values are represented as mean ± SE (circle and bars, respectively) and as individual values (point). Mating status is indicated by point fill and shape.

RNAi treatment had a significant effect on the average nuclei area (ANOVA; *F *= 91.33, df = 1,45, *P *< 0.001; Fig. 4). Mating status did not have a significant effect on the average nuclei area (*F *= 0.92, df = 1,45, *P *= 0.34; Fig. 4). There was a significant interaction between RNAi treatment and mating status on the average nuclei area (*F *= 4.09, df = 1,45, *P *= 0.05; Fig. 4). Overall, embryos from *dsDnmt1*-fed females had significantly smaller nuclei than those from *dseGFP*-fed females.

## Discussion

In this study, we showed that DNMT1 was required for early embryogenesis in *B. tabaci* using RNAi knockdown (*dsDnmt1*) and examined how it effects life history traits in both haploid and diploid offspring. We also examined and characterized early embryogenesis and documented timing of effects of *Dnmt1* knockdown. Because virgin females only produce male offspring and mated females produce offspring of both sexes, comparing fecundity and offspring mortality between virgin and mated females can determine if differential mortality based on sex is occurring (Bondy and Hunter 2019b). Our findings for life-history effects mirrored earlier studies of whiteflies (Cunningham et al. 2024) and the milkweed bug *O. fasciatus* (Bewick et al. 2019, Amukamara et al. 2020, Washington et al. 2021), but here we examined both mated- and virgin-produced offspring. We found a statistically significant reduction in the number of eggs laid by *dsDnmt1* females. This did not depend on whether the eggs were produced from virgin or mated females. Likewise, *dsDnmt1* treatment reduced egg viability of both mated- and virgin-produced eggs. However, these were not sex-specific effects, as the adult sex ratio in offspring from mated females was not statistically significantly different among treatments. Virgin females produced only male offspring as expected. The early life-history effects of *Dnmt1* do not appear to be sex-specific or ploidy-dependent based on the measured traits.

We also catalogued key events in *B. tabaci* embryonic development, for which there are no detailed embryological studies published. Based on our descriptions of the timing and morphogenesis during *B. tabaci* embryogenesis, our results indicate that the development of this species is comparable to other insect systems such as *O. fasciatus* (Liu and Kaufman 2004, Panfilio et al. 2006), *Gryllus bimaculatus* De Geer (Donoughe and Extavour 2016), *Murgantia histrionica* Hahn (Hernandez et al. 2020), *Nilaparvata lugens* Stål (Fan et al. 2020). This suggests the pattern of development may be conserved among hemipterans and perhaps hemimetabolous insects in general. Based on the timing of developmental stages in embryos from both virgin and mated females, our study indicates that sex does not affect developmental timing. This result aligns with what has been observed in the hymenopteran *Nasonia diterpenes* (Walker), another obligate haplodiploid system, but one that is holometabolous (Arsala and Lynch 2017), suggesting that this phenomenon may be a widespread feature of embryonic development in obligate haploid systems.

Finally, we used *dsDnmt1* knockdown treatments compared to controls to examine timing and potential function of DNMT1 during embryogenesis. Knockdown of *Dnmt1* mRNA, and the subsequent reduction in the DNMT1 protein (Shelby et al. 2023), prevented *B. tabaci* embryos from forming a germ rudiment. Cleavage may also have been affected as loss of *Dnmt1* resulted in smaller nuclei. In other insect taxa, loss of *Dnmt1* has resulted in similar embryo failure during the blastoderm phase or before gastrulation (Schulz et al. 2018, Ventós-Alfonso et al. 2020, Arsala et al. 2022). This suggests that DNMT1 plays an evolutionarily conserved role specifically in early embryogenesis in insects. Indeed, expression of *Dnmt1* peaks during early embryogenesis before gastrulation and decreases as development progresses (Arsala et al. 2022). Also, given that loss of *Dnmt1* affected embryos of both sexes before the blastoderm phase, it is likely that ploidy (and therefore amount of DNA) may not dictate DNMT1 function. The timing during which embryonic development is deterred during *Dnmt1* knockdown, in addition to the lack of evidence for ploidy-driven differences in *Dnmt1 knockdown* embryo development, suggests that DNMT1 may function similarly to a maternal factor and may be regulated by the timing of the maternal clock. The timing and phenotype of the *Dnmt1* knockdown embryos could indicate that these embryos are not capable of completing either the transition from maternal to zygotic transcription or the mid-blastula transition, a stage in development characterized by changes in the cell cycle and loss of synchronous cell divisions (Vastenhouw et al. 2019). We indirectly observed cell cycle changes via the changes in nuclei size observed during knockdown. However, future studies are needed to decipher how DNMT1 interacts with DNA and other components to understand its mode of action. Future studies will also need to examine transcriptions patterns of *Dnmt1* during embryonic development in order to determine if DNMT1 functions as a maternal factor.
